# Depression and Anxiety Symptoms Relate to Distinct Components of Pain Experience among Patients with Breast Cancer

**DOI:** 10.1155/2012/851276

**Published:** 2012-11-21

**Authors:** Sarah K. Galloway, Megan Baker, Pierre Giglio, Steve Chin, Alok Madan, Robert Malcolm, Eva R. Serber, Sharlene Wedin, Wendy Balliet, Jeffrey Borckardt

**Affiliations:** ^1^Division of Biobehavioral Medicine, Department of Psychiatry & Behavioral Sciences, Medical University of South Carolina, 67 President Street, 1-South, Charleston, SC 29425, USA; ^2^Division of Surgical Oncology, Department of Surgery, Medical University of South Carolina, 67 President Street, 1-South, Charleston, SC 29425, USA; ^3^Division of Hematology and Oncology, Department of Medicine, Medical University of South Carolina, 67 President Street, 1-South, Charleston, SC 29425, USA

## Abstract

Breast cancer is a leading cancer diagnosis among women worldwide, with more than 210,000 new cases and 40,000 deaths per year in the United States. Pain, anxiety, and depression can be significant factors during the course of breast cancer. Pain is a complex experience with sensory, affective, and cognitive dimensions. While depression and anxiety symptoms are relatively common among breast cancer patients, little is known about the relation between these psychiatric factors and distinct components of the pain experience. In the present study 60 females presenting to an NCI-designated Cancer Center with newly diagnosed breast cancer completed the Center for Epidemiological Studies 10-item Depression Scale, the State Instrument of the Spielberger State-Trait Anxiety Inventory, and the McGill Pain Questionnaire. Findings indicate that anxiety and depression are common among newly diagnosed breast cancer patients; furthermore, patients experience an appreciable amount of pain even before oncologic treatment starts. State anxiety serves as a predictor of the sensory dimension of the pain experience, whereas depression serves as a predictor of the affective dimension of the pain experience.

## 1. Introduction

 Breast cancer is a leading cancer diagnosis among women worldwide, with more than 210,000 new cases and 40,000 deaths per year in the United States [1]. Pain can be a complex and significant factor during the course of breast cancer and is often related to the underlying malignancy and tumor burden, surgeries, chemotherapies, radiation, and hormonal therapies used to treat the malignancy. Notably, pain is usually not a symptom of tumor burden in less advanced disease and is only experienced in 5–15% of breast cancer patients prior to diagnosis [2].

 Contemporary models have expanded pain to involve more than just a nociceptive experience. Melzack and Casey [3] describe a multidimensional model of pain processing with sensory, affective, and cognitive components. Sensory aspects help to identify the location, size, and intensity associated with the noxious stimuli, whereas affective components of pain refer to the unpleasantness or distress associated with the physical sensation of pain. Lastly, cognitive components include evaluation and interpretation of the meaning of the pain experience. 

Pain, anxiety, and depression often co-occur [4]. Research indicated that individuals with pain endorse significantly elevated rates of depression and anxiety when compared to those without pain. More specifically, results of epidemiological studies across 17 different countries suggest that anxiety disorders are 2-3 times more prevalent in chronic pain populations [5]. Results from a nationally representative sample indicate that both depression and anxiety are 1.5 times to 4 times more likely in individuals with pain conditions compared to those with no pain condition [6].

Anxiety and depression commonly occur in cancer patients who are facing multiple biological and psychosocial stressors. Biologic stressors impacting an individual's function include the tumor burden, treatment morbidity, neurobiological changes, pain, and physical sensations. Psychosocial stressors impacting an individual's functioning include themes of uncertainty, loss of control, changes in life trajectory, and increased dependency, as well as changes in role functioning, appearance, and identity [7]. Anxiety and depression can develop at different points on the treatment continuum from the point of abnormal finding to diagnosis, initiation or completion of treatment, progression of disease, survivorship, and throughout palliative care [8]. 

When compared to the general population, cancer patients are at higher risk for experiencing depression and anxiety. Within the general population, the 12-month and lifetime prevalence rates for major depressive disorder are 6.6% and 16.5% [8, 9], whereas the prevalence rates for major depressive disorder within the cancer population are 0–38% [10]. The prevalence rate increases up to 58% of the cancer population when considering other depressive disorders [10]. Within the general population, the 12-month prevalence rate of anxiety is 8%. As with depression, anxiety is more prevalent in the cancer population with epidemiologic studies estimating rates at 10–30% [11]. Burgess et al. [12] found in their study with early-stage breast cancer patients that 33% of their sample was diagnosed with depression and anxiety at cancer diagnosis, 15% at 1 year after cancer diagnosis, and 45% at cancer recurrence. Similarly, Vahdaninia et al. [13] found in their recent prospective study of breast cancer patients that 47.4% of patients experienced severe anxiety, and 18.0% experienced clinically significant symptoms of depression at a prebreast cancer diagnostic assessment. Interestingly, at 18-month followup mean levels of anxiety and depression had decreased, but 38.4% of patients still experienced severe anxiety, and 22.2% experienced clinically significant depression. 

 Specifically within the breast cancer population, Vahdaninia et al. [13] examined the relation between anxiety, pain, and depression at baseline prediagnosis, 3-month follow-up, and 18-month follow-up. Researchers did not find a significant relation between anxiety and depression and pain at 3-month follow-up, but at the 18-month follow-up pain was significantly related to both anxiety and depression. Moreover, pain was the most significant variable related to anxiety at 18-month followup over and above disease state. 

 In their systematic review of cancer pain and depression, Laird et al. [14] found that pain and depression were highly prevalent. They additionally found that certain pain characteristics were related to depression. First, pain intensity [15–17] and pain duration [16, 18] were associated with increased levels and risk of depression respectively. Next, when responding to the McGill Pain Questionnaire, depressed patients had higher affective pain intensity scores and used a greater number of affective pain descriptors when compared to the nondepressed patients in the sample [17].

The current study seeks to describe the pain experience of women recently diagnosed with cancer who have not yet undergone cancer-related treatment. We focus on the prevalence of anxiety and depression and their relation to multiple dimensions of pain. In addition to describing anxiety, depression, and pain within this unique sample, we strive to build on the existing literature by examining whether anxiety and depression differentially predict components of the pain experience. 

## 2. Methods and Materials

### 2.1. Participants and Procedure

 In the present study 60 females presenting to a southeastern NCI-designated Cancer Center with newly diagnosed breast cancer (prior to treatment) completed paper-and-pencil measures in the waiting room. Hospital staff then entered the scores from the measures into a computer system. The data were collected as part of routine clinical practice, and IRB approval was attained in order to permit publication of the data in aggregate for the present study. A chart review provided participants' demographic information. 

### 2.2. Measures

#### 2.2.1. Depression

 The Center for Epidemiological Studies 10-Depression Scale (CESD-10) [19] is a ten item self-report scale measuring somatic, affective, cognitive, and interpersonal components of depression. Respondents report on the intensity that they experienced symptoms over the past two weeks using a 4-point scale (1 = rarely or none of the time;  4 = all of the time). Sample items include: “I felt depressed” and “I felt that everything I did was an effort.”

#### 2.2.2. Anxiety

The State-Trait Anxiety Inventory-State Scale (STAI-S) [20] is a 20-item self-report scale measuring worry, tension, and apprehension that the respondent experiences in his or her current circumstances (state anxiety). Respondents report on the frequency that they experience symptoms on a 4-point scale (1 = not at all; 4 = very much so). Sample items include: “I feel nervous and restless” and “I feel that difficulties are piling up so that I cannot overcome them.”

#### 2.2.3. Pain

The McGill Pain Questionnaire-Short Form (SF-MPQ) [21] is a self-report scale measuring qualitative and quantitative components of pain. The scale consists of 15-descriptor items with 1–11 describing sensory pain dimension and 12–15 describing affective pain dimensions. Items are then ranked on a 4-point scale (0 = no pain to 4 = severe pain). Descriptor items include: “throbbing,” “shooting,” and “stabbing.”

### 2.3. Data Analyses

Descriptive statistics were calculated for all study variables. Simple regression was utilized to address the predictive relationships between anxiety, depression, and components of pain. Pairwise deletion was utilized to account for missing data. 

## 3. Results

 Six percent of the sample was between the ages of 20 and 29, 11% was in the 30–39 age-range, 12% was in the 40–49 age range, 24% was in the 50–59 age range, 29% was in the 60–69 age range, and 18% of the sample was 70 or older. 72% of the sample was Caucasian, 27% was African American, and 1% was of Asian descent. 1% of the sample identified themselves as being of Hispanic ethnicity.

 Seventy-two percent of participants exceeded the cutoff for clinically significant anxiety symptoms on the STAI (mean  score = 46.75, SD = 6.14), and 48% exceeded the cut-off for clinically significant depression on the CESD (mean  score = 10.25, SD = 5.83). Mean percent of total possible sensory and affective pain scores from the MPQ were 44% and 45%, respectively, suggesting a moderate amount of reported pain by respondents despite not yet starting cancer treatment.

**Figure 1:**
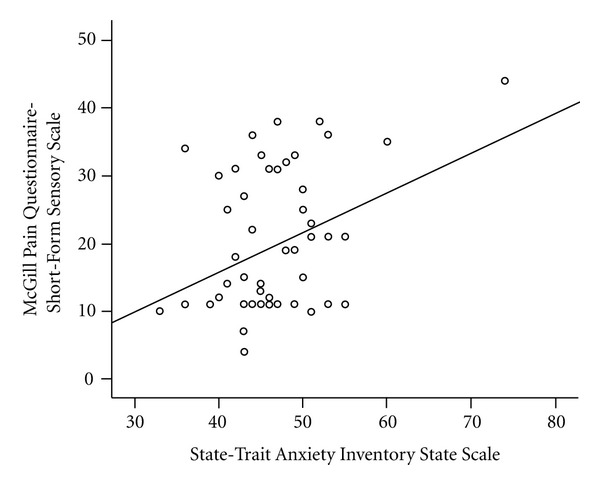
Scatterplot representing the relation between state anxiety and sensory pain.

**Figure 2:**
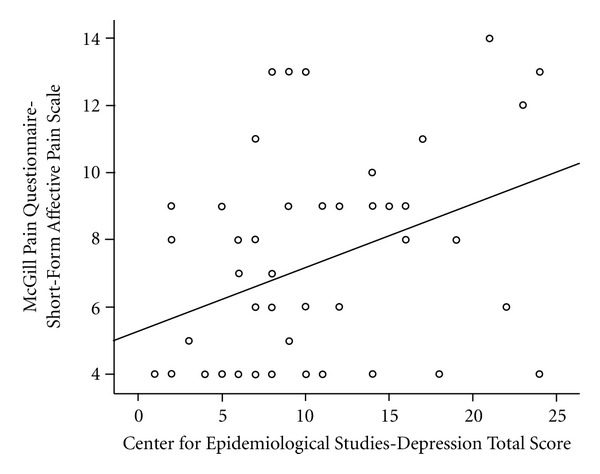
Scatterplot representing the relation between depression and affective pain.

 Anxiety scores were positively predictive of the sensory component of the pain experience (*r*(58) = .36, *P* = .006) but not the affective component of the pain experience (*r*(58) = .12, *P* = .36), whereas depression scores were predictive of the affective component of the pain experience (*r*(58) = .36, *P* = .005) but not the sensory component (*r*(58) = −.04, *P* = .73).  Figure 1 represents the relation between anxiety and sensory pain, and Figure 2 represents the relation between depression and affective pain. Anxiety scores and depression were not related (*r*(58) = −.004, *P* = .97).

## 4. Discussion 

This study examined the prevalence and relation between anxiety, depression, and pain among newly diagnosed breast cancer patients. We found that 72% of participants exceeded the clinical cut-off for anxiety, and 48% exceeded the clinical cut-off for depression. Additionally, although participants had not yet started oncologic treatment, they reported experiencing moderate amount of pain. Interestingly, the relation between psychiatric factors and components of pain differed. Increased levels of state anxiety were significantly related to increased levels of the sensory pain component but were not related to the affective pain component. On the other hand, increased levels of depression were significantly related to increased levels of the affective pain component, but were not related to the sensory pain component. 

Findings from this preliminary study suggest that anxiety and depression may be common among newly diagnosed breast cancer patients, and that these patients may be experiencing an appreciable amount of pain even before oncologic treatment starts. In the current study, increased levels of pain experienced by women could be related to recent biopsy, primary tumor burden, and metastatic disease. Furthermore, findings suggest that state anxiety may be a predictor of the sensory dimension of the pain experience, whereas depression may be a predictor of the affective dimension. 

Results from the current study are consistent with previous research finding that clinically significant anxiety and depression are common within the newly diagnosed cancer population. More specifically, rates of depression were similar to previous research given our use of the CES-D to identify depressive symptomatology. The CES-D is a screening device for symptoms rather than a diagnostic tool; consequently, prevalence rates of clinically significant depression are reflective of depressive diagnoses across the continuum-adjustment disorders with depressed mood to major depressive episodes. Rates of anxiety were significantly higher in our study than in previous research and could be reflective of our methodology in measuring anxiety prior to participant's first meeting with their oncologist. Anxiety related to cancer is typically experienced at high rates and intensity until a treatment plan has been established. Once a treatment plan has been developed, some women experience a decline in state levels of anxiety. 

As per the relation between psychiatric factors and components of pain, this study validates previous findings on a strong relationship between depression, anxiety, and pain within a cancer population. Furthermore, our study replicates findings from Sist et al. [17] indicating that depression is related to the affective dimension of pain within the cancer population. The current study findings expand on previous studies within the cancer population to demonstrate that anxiety and depression are differentially related to the affective and sensory components of pain.

 As the current study did not assess for underlying medical conditions outside of cancer contributing to the pain experience, we cannot attribute pain solely to tumor burden or biopsy. Staging and cancer-related information is additionally necessary for helping to establish an etiology to the sensation component of the pain experience. Our correlational study provides preliminary findings to the relationship between different psychiatric conditions and different components of the pain experience. Although we cannot extrapolate to the underlying causality of the relation between psychiatric factors and cancer patients' pain experience, our study provides evidence for future longitudinal studies to examine the temporal course of pain, depression, and anxiety and coregulation of these factors across the continuum of cancer diagnosis, treatment, and survivorship. Future epidemiological, neuroimaging, and interventional research may be warranted to better understand these patterns and to determine optimal strategies to tailor interventions targeting anxiety, depression, and pain among breast cancer patients. Current study findings have implications for the treatment of pain within the breast cancer population. Clinicians should attend to the multidimensional nature of the pain experience and the high comorbidity between pain and anxiety and depression. Furthermore, in order to fully address and treat pain, clinicians should take a multidisciplinary and integrative approach attuned to sensory and affective components.
